# Is student mentoring career-defining in surgical disciplines? A comparative survey among medical schools and medical students for mentoring programs

**DOI:** 10.3389/fmed.2022.1008509

**Published:** 2022-11-23

**Authors:** Stefan Ferdinand Hertling, David Alexander Back, Britt Wildemann, Ekkehard Schleußner, Mario Kaiser, Isabel Graul

**Affiliations:** ^1^Department of Obstetrics, Jena University Hospital, Jena, Germany; ^2^Department of Orthopaedics, Jena University Hospital, Eisenberg, Germany; ^3^Dieter Scheffner Center for Medical Education and Educational Research, Charité—Universitätsmedizin Berlin, Berlin, Germany; ^4^Department of Trauma, Hand and Reconstructive Surgery, Jena University Hospital, Friedrich Schiller University Jena, Jena, Germany; ^5^Module Integration Optics, Jenoptik Light and Optics Division, Jena, Germany

**Keywords:** mentoring, medical students, gender, career, surgery

## Abstract

**Objective:**

Facing a shortage of young surgeons, this study aimed to examine the availability of mentoring programs and if this can counteract this lack.

**Summary background data:**

Medical mentoring programs have proven to be decisive to influence students’ later career decisions. Since their structure may depend on the medical school and the effort of single disciplines, the offers are often very heterogeneous.

**Methods:**

Anonymous online-questionnaires were developed and distributed among medical students in Germany and the dean for teaching of the medical schools from July 2019 to January 2020 in Germany. Data of the availability of mentoring programs, their structure and the impact of surgery were collected.

**Results:**

Forty three medical schools participated, with 65% offering mentoring programs. 18 of medical schools had no additional funding available for this. Surgical subjects participated in these programs in only 30%. Additionally, 1,516 medical students participated in the second survey. A total of 70% had already participated in a mentoring program with a significantly higher proportion of men. Of these, 94% stated that this was helpful and had an impact on their career planning, without any gender differences. 95% would participate in structured surgical mentoring programs and 95% agreed that this could have an impact on their career planning.

**Conclusion:**

Mentoring programs may be able to influence career planning, nevertheless participation by surgical specialties has been low. Becoming more active in providing mentoring programs with a special focus on women and offering more surgical content can be a way to counteract the lack of surgical trainees.

## Introduction

Factors influencing medical students in their choice of later specialization are not yet well understood. Even at the very end of their studies, there are still quite a few medical students without a clear choice of specialization (1). However, the current generation of students in particular attaches importance to the compatibility of family, career, and the workload expected in the future (2). All the more, it seems that the discussion of student recruitment during clinical training in their education is coming to the fore. One way to arouse the students’ interest in different fields of study could be created and intensified with mentoring programs.

Mentoring programs in medical schools exist to provide students with support and guidance. Although the definition of mentorship varies, it is typically described as a relationship between a senior (mentor) to a junior person (mentee) to reflect career development, professional growth or satisfaction (3, 4). Nevertheless, the availability and structure of mentoring programs for medical students internationally, Europe-wide and nationally in Germany remains heterogeneous and confusing. Data from Germany published by Meinel et al. 10 years ago showed 22 mentor programs in German medical universities (5). No newer data have been collected since this study. Thus, the current situation for the proposal of mentoring programs in medical education in Germany is currently unclear, while also the international data situation is relatively low. An overview of published reviews of mentoring programs among medical students is shown in Table 1.

**TABLE 1:** Review articles searched by “mentoring” and “medical students” in PubMed on 11/01/2020.

References	Year	Main statement	Number of included articles
Skjevik et al. (41)	2020	Group mentorship programs benefit from being longitudinal and mandatory. They should provide regular meetings where discussions and personal reflection occur.	20
Chua et al. (42)	2020	In mentoring programs, there was a need for balance between ensuring consistency and flexibility to meet the individual needs of stakeholders throughout the stages of the mentoring process.	71
Guraya and Abdalla (43)	2020	Peer-assisted learning can be used as a valuable learning tool in the medical field.	11
Radha Krishna et al. (44)	2019	Role modeling, teaching and tutoring, coaching and supervision lie within a mentoring spectrum of increasingly structured interactions, assisted by assessments, feedback and personalized support that culminate with a mentoring approach.	104
Farkas et al. (45)	2019	Mentoring programs for medical students can positively improve medical school satisfaction and career development.	30
Nimmons et al. (46)	2019	Outline of the challenges encountered, potential benefits, and critical future implications for mentees, mentors, and institutions.	82
Tan et al. (47)	2018	There were two vital elements of an effective mentoring framework: flexibility and structure	34
Burgess et al. (48)	2018	Mentoring had an essential influence on personal development, career guidance and career choice.	not named
Frei et al. (49)	2010	Mentoring was a career advancement tool for medical students.	25
Buddeberg-Fischer and Herta (50)	2006	Despite promising results, no publication contained statements on the effectiveness or the efficiency of the programs.	16

Of the 346 articles found, ten could be included. Inclusion criteria were review articles, where medical students were addressed with mentoring programs during medical school. The main statements of the reviews were mentioned. Nine reviews point out the importance of mentoring programs and try to evaluate important facts for the successful program. One review criticizes the lack of information on the effectiveness and efficiency of the programs.

Declining interest in surgical careers has been observed for more than a decade (6). In addition, the community of medical professionals is aging (7). However, US data showed that while 45% of first-year medical students were interested in a surgical career, only 7% of graduating students were matched to surgical residences (8). Next to personal skills and experiences, the work-life balance becomes more and more important for young medical students (2, 9) rather incompatible with a surgical career. In addition, the presence of the mentor is very essential to 90% of the questioned students (10). Successful mentoring programs can lead to the strengthening of interests in the area concerned which in turn help to initiate a career decision. This is especially important as, the surgical specialties are facing a shortage of junior staff in Germany (11, 12).

The first purpose of the study was to assess the current situation of mentoring programs offered at medical schools from the perspective of medical schools and medical students.

On the one hand medical schools were evaluated for their range of mentoring programs and their structure, initiators, and funding. On the other hand, medical students were asked about their experiences with mentoring programs in their studies.

The second aim was to determine the current career planning of the students and to check whether the students feel that this can be changed through surgical mentoring programs.

## Materials and methods

### Questionnaire design

The study questionnaires were designed concerning published guidelines on questionnaire research in a web-based design (13). The selection of questions for the questionnaires were based both on comparable work and on the quality criteria for online questionnaires (14). The surveys were created in SurveyMonkey™ (SurveyMonkey, San Mateo, CA). Both questionnaires are included as additional material.

### Performance of the surveys

The students’ survey was distributed among all medical student councils of the 36 medical schools in Germany. The duration of the survey was from July 2019 to January 2020. Medical students at all stages of their studies were included. In Germany, the study program is divided into 3 sections, the first 2 years of pre-clinical basic studies, 3 years of clinical studies and a practical year in the clinic. With a population of 98,736 medical students, a confidence interval of 95% and an error margin of 2.5, the target case number was 1,513. Thus the online survey can be considered representative of the entire German medical student population.

The questionnaire was distributed *via* e-mail distribution lists of the student councils to all enrolled students. In an information letter, participants were informed that their data would be treated strictly confidential and anonymized. Access to the study was granted with a survey link and a QR (quick response) code in the cover letter. The responsible local ethics committee was informed and had no objections to the study.

The medical school survey was distributed among all deanery of the 50 medical universities of German-speaking medical schools in Germany, Austria and Switzerland between October till December 2020 *via* email. In an information letter, participants were informed that their data would be treated strictly confidential and anonymized. Access to the study was granted with a survey link and a QR code in the cover letter.

### Medical school questionnaire contents

A 12-item, self-administered online questionnaire survey was developed according to the students’ questionnaire. The main sections were:

1. *Existence and claims*: Number of students enrolled, offers of mentoring programs and their use of these by students
2. *Structure of the mentoring programs*: disciplines involved, specifications for the structure, Requirements for the structures and specifications for the structure and content of the program, possible orientation toward defined standards of the German Medical Association
3. *Finances and support*: Funding of mentoring programs, desire of medical schools for support from professional societies or academic institutions
4. *Demand*: assessing the potential uptake of mentoring programs by students if they were offered.

### Students questionnaire contents

A 10-item, self-administered online questionnaire survey was developed based on a comprehensive list of questions bases on the published research in mentoring among medical students. Members of the Teaching Working Group of the DGOU (German Society of Orthopaedics and Traumatology) Young Forum were invited for the validation process to provide feedback on question format, comprehensiveness, clarity, and flow (15). According to this, the questionnaire was refined. It consisted of five binominal questions and five multiple-choice questions and was entitled “mentoring programs for a surgical career.” The main sections were:

1. *Epidemiological demographics*: gender and study level.
2. *Mentoring relationships:* Participation in a mentoring program in the past, benefits from the mentoring program, positive encouragement of choice of specialization
3. *Career specialization:* Desired subject choice, if surgical, which specific discipline
4. *Mentoring relationship in surgery:* Wish for participation in a structured mentoring program in surgery, assumption of the positive influence on the choice of a surgical specialty

The aim was a short duration of the survey of maximum of 3 min to keep the drop-out rate as low as possible and the motivation to answer the questions as high as possible (16). Also, open questions were avoided, as this can also have a demotivating effect on the participants.

### Data analysis

Only fully completed questionnaires were included in the subsequent analysis. Analysis of results was undertaken using SurveyMonkey™ and the Statistical Package for the Social Sciences, SPSS (version 17.0, SPSS Inc., Chicago, IL, USA). *P*-values were calculated using the Mann-Whitney *U*-test. A *p*-value of less than 0.05 was considered to be significant.

The responsible local ethics committee was informed and had no objections to the study (Reg.-Nr.: 2019-1456-Bef). All experimental protocols were approved by the local ethics committee of the University Jena. The informed consent was obtained from all participants.

The study was carried out in accordance with the relevant guidelines and regulations.

## Results

### Medical school questionnaire

Of the 50 German-speaking universities that offer medical studies, 86% (43/50) took part in the survey.

### Existence and claims

The majority (51%; 22/43) of universities had 2,000–4,000 students of human medicine, 35% of the universities had less than 2,000 students and 14% had more than 4,000 students. 65% (28/43) of the universities offered a mentoring program for students, 12% (5/43) said they planned to offer one in the future and 19% (8/43) had offered a program in the past but no longer did so. Only two of the universities negated the question if they considered a mentoring program. The acceptance of such programs by the students was mixed. An average of 200–500 students per medical school was indicated to take part in a mentoring program. Overall, approximately 20% (16,400/82,050) of medical school students participated in a mentoring program.

### Structure of the mentoring programs

Seventy two percent (31/41) of the universities described their planned, past or current mentoring program to be structured, with 71% (29/41) referring to defined individual criteria regarding the structure, content and organization of the mentoring program. Only 29% (12/41) of the universities adhered to the standards of the German Medical Association. A total of 37% (15/41) offered their mentoring program across clinics. Surgical subjects participated in the mentoring programs in 46% (13/28).

### Finances and support

Funding is provided by 43% (18/42) without an existing budget. A total of 9% (4/42) medical schools had up to 5,000 Euros, 29% (12/42) between 5,000 and 10,000 Euros, 7% (3/42) between 10,000 and 20,000 Euros and 7% (3/42) 20,000–50,000 Euros, and 5% (2/42) over 50,000 Euros as budget for mentoring programs. Whether medical schools wanted more support from professional societies or from academic institutions, 45% (19/42) answered yes. One medical school did not answer the questions about funding.

### Demand

All 14 medical schools that do not currently offer a mentoring program saw students taking part of any offered mentoring program.

### Students questionnaire

Of the 98,736 medical students enrolled at German medical schools (in 2019/2020), 1,516 responses could be received. This corresponded to a percentage of 1.54%.

### Epidemiological demographics

Overall, 55% participants were male (*n* = 653), female (*n* = 840) and divers (*n* = 23). Responses were received from medical students in early semesters (first 2 years of studying pre-clinical medicine) in 10% (*n* = 156), from the later semesters (3 years of studying clinical medicine) in 35% (*n* = 535) and from the practical year 42% (*n* = 634), 13% (*n* = 191) were not defined (pregnancy, vacation semester, PhD thesis).

**FIGURE 1:**
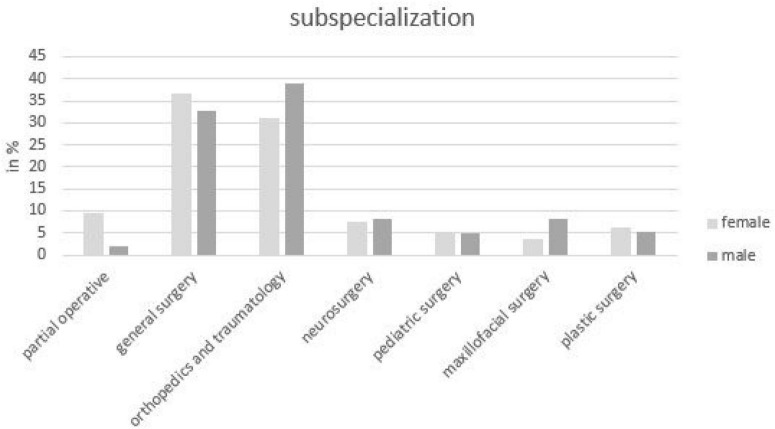
Epidemiological breakdown of the questionnaire replies. The data were given in percent, subdivided by gender and semester level (*n* = 1,516).

In the pre-clinical semester significantly more female students (*f* = 89, *m* = 67; *p* < 0.001) answered the questionnaire and in the practical year more male students (*f* = 241, *m* = 382; *p* < 0.001). For the clinical part, an equal distribution was observed (*f* = 246, *m* = 280; *p* = 0.54). Participation increased as students reach a higher level of education (Figure 1).

For further analysis diverse gender (*n* = 23) was specified but not statistically evaluated due to the small numbers to keep the clarity.

### Mentoring relationships

**FIGURE 2:**
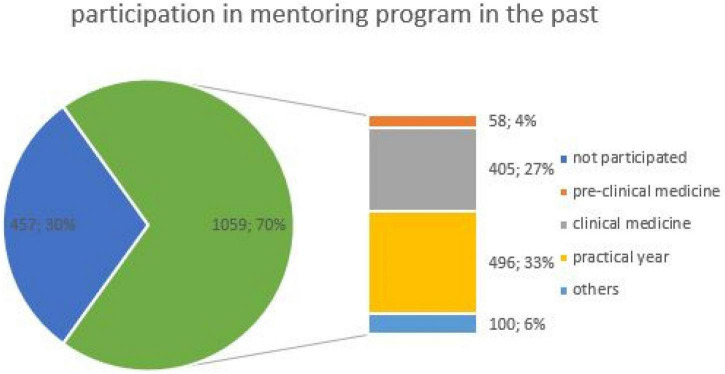
Distribution of students who had participated in the mentoring program in the past in absolute numbers (*n* = 1,516) and in percent. The figure is divided according to the participation mentoring program in the pie chart (blue: not participated, green: participated) and divided by study period in the bar chart.

The majority (70%, 1,059/1,516) of those surveyed had a mentor-mentee relationship in their previous studies (Figure 2). Significantly more male students than female ones had taken part in a mentoring program in their studies (*f* = 415, *m* = 622; *p* < 0.001). In the higher semesters, the number of students who had attended a mentoring program was significantly higher (pre-clinical = 58, clinical = 396, practical year = 486, other = 97; *p* < 0.001). In the practical year, 70% (168/241) of the female and 83% (318/382) of the male students had participated in such a program.

Of the responses with a mentoring relationship, 94% (993/1,059) considered this as helpful with a personal benefit and 94% (1,000/1,059) confirmed an influence on the later choice of career specialization through the mentoring relationship. There was no significant difference in gender (*f* = 392, *m* = 601; *p* = 0.61), but in study period (pre-clinical = 53, clinical = 386, practical year = 487, other = 89; *p* = 0.01).

Students of pre-clinical medicine (5%; 5/58) tended to deny that the mentoring program influenced on their later careers. Only 3% (37/1,059) did not see any connection between participated mentoring programs to their career specialization. Higher semester students (98%; 488/496) saw an influence on their choice of subject area.

### Career specialization

A total of 77% (1,161/1,516) of the responders were interested in a career in a surgical specialization. Interestingly, there was no difference in gender for choosing a surgical career (*f* = 500, *m* = 645; *p* = 0.87). Study period had no significant influence on surgical career choice, too (pre-clinical = 121, clinical = 414, practical year = 486, other = 140; *p* = 0.19).

Subdividing the surgical career opportunities, 96% (1,097/1,161) voted for surgery and 4% (47/1,161) for specializations with partial surgical care like gynecology, urology or otorhinolaryngology. Gender had an important influence. Female students wished to choose disciplines with partial surgical care and male rather wished to choose surgical careers (*f* = 34 *m* = 12; *p* < 0.01). In this question, the study period within the medical education program showed no significant influence (pre-clinical = 7, clinical = 19, practical year = 17, other = 4; *p* = 0.295).

**FIGURE 3:**
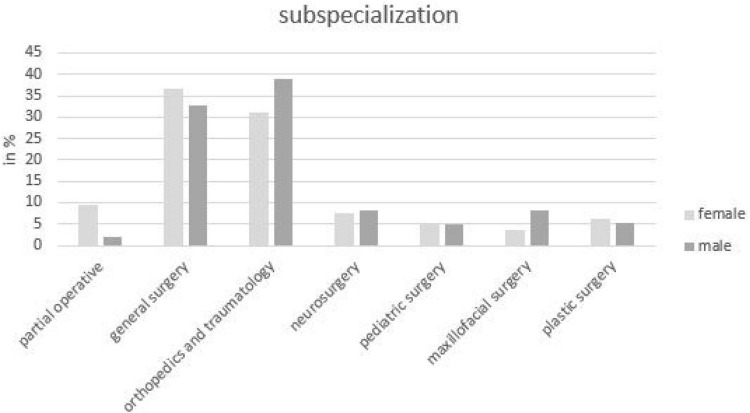
Distribution of the sexes in the surgical subjects in percent (absolute numbers) of all students, which want to choose a surgical subject area (*n* = 1,161).

Surgery was divided into general surgery, orthopedics and traumatology, neurosurgery, pediatric surgery, maxillofacial surgery, and plastic surgery. Most students were interested in orthopedics and traumatology (37%) and general surgery (35%). Looking at the percentage distribution of sexes of the most strongly represented surgical disciplines individually, more female students wanted to go into general surgery (42%; 189/451) and more male students into orthopedics and trauma surgery (40%; 250/633) (Figure 3).

### Mentoring relationship in surgery

**FIGURE 4:**
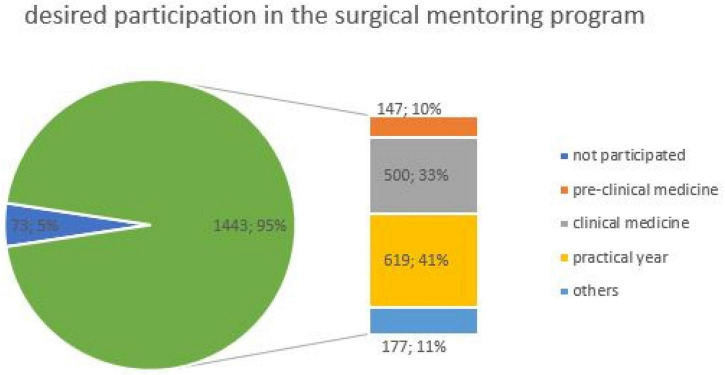
Distribution of students who wished to participate in a surgical mentoring program (*n* = 1,516). The figure is divided according to the participation mentoring program in the pie chart (blue: not participated, green: participated) and divided by the study period in the bar chart.

When asked about possible participation in a surgical mentoring program, 95% (1,443/1,516) of the responders showed interest in such a program with the significant difference in gender (*f* = 607, *m* = 816; *p* < 0.001) and study period (pre-clinical = 147, clinical = 500, practical year = 619, other = 177; *p* = 0.04) (Figure 4).

The higher semester would take more frequently part in surgical mentoring programs. Male students (97%; 816/840) would be more likely to participate in a surgical mentoring program than female students (93%; 607/653). Additionally, 95% (1,438/1,516) thought, a mentoring program could influence their choice toward a surgical specialization with significant difference in gender (*f* = 607, *m* = 811; *p* < 0.001) or study period (pre-clinical = 146, clinical = 502, practical year = 615, other = 175; *p* = 0.04). Only a remarkably small proportion of students 9 (4 female, 5 male) denied any expected effect on a surgical career choice by a mentoring program.

## Discussion

As a multicenter survey this study aimed to provide an overview of presence and participation in mentoring programs. The study focused on questions from medical schools and students about the availability and use of mentoring programs.

When mentoring programs are available for students, this could influence the choice of specialization for surgical specialties (17). Knowledge about the impact of mentoring programs in medical undergraduate education could be used to guide young people also into disciplines with a shortage of young talents. To the authors’ knowledge, this is the first representative study dealing with the current mentorship availability and at the same time included the choice of surgical career specialization in the consideration in Germany.

Currently, about two-thirds of German-speaking medical schools offer mentoring programs. This results in 1 in 3 students not even having the opportunity to decide if they want to participate in a mentoring program in 2020. Although the remaining one-third of medical schools estimated that this would be well received by students, eight of the medical schools terminated existing mentoring programs. The number of participating students in the offered ones was low compared to the number of students enrolled at the single institutions. On average, 20% of students participated in a mentoring program. The question of whether there were simply not more places for mentees, or no student interest in this, cannot be answered by the study. It is striking, however, that of the 15 medical schools without offering mentoring programs, nine have no budget for it. The question is whether providing more financial resources could significantly increase the opportunities for mentoring programs.

A wide gap is emerging between the answers of universities (20% participation rate of students) and medical students (70% took part in a mentoring program). While the medical school survey reflects the more objective situation, a selection bias in the student survey may exist, since especially students who were already familiar with the topic of mentoring participated. Also the different definition as an objectifiable mentoring relationship to rather loose mentorships, which are difficult to trace on the part of the medical school, could play a role. As a sign of this bias, 71% of medical schools offer structured mentoring programs, as the majority of more loose mentor-mentee relationships are not reflected here. But the supply of possible mentee places is certainly also limited, so that even in existing mentoring programs not everyone with the desire to participate can do so. Furthermore, it is noticeable that only in 30% (13/43) of the medical schools surgical subjects were involved in mentoring programs, revealing an objective lack in surgical engagement in this topic. This development is particularly deficient in light of the growing shortage of young surgeons.

In the western world, high workload and low work-life balance are described as the main reasons why medical students do not plan a surgical career (2, 9, 18, 19). In contrast to the study of Kleinert et al., who stated that 11% of female and 19% of male German medial students planned a surgical career (12), 77% of the medical students in this study wanted to pursue a surgical career. It is likely that especially students interested in surgery participated in the survey, and even insecure students were more likely to choose a surgical specialty as this could be suggested by the questionnaire.

Analyzing the online survey in terms of students’ gender, under-representation of women (45%) in the study becomes apparent and additionally the percentage of women decreased during the study periods. However, since females are with up to 70% much stronger represented among medical students, an equal distribution is still an under-representation of women (20). Women in particular showed significantly lower participation in mentoring programs in the past despite high interest in surgical mentoring programs, if they could decide. The study also showed that women were as likely as men to pursue a surgical career with a significantly more frequent decision to pursue disciplines with partial surgical care.

In literature, women are still a minority in surgery, accounting for 28% of surgeons in the USA (21) and 18% in Germany (20). The reasons for this were sexual harassment and gender discrimination (22–24), perceptions about the challenges of a surgical lifestyle, a lack of parental leave or childcare (22, 23, 25) and a lack of role models or mentorship (22–24, 26–29) in literature.

Interviewed at the Ruth Jackson Orthopedic Society about reasons for choosing an orthopedic specialty, female surgeons stated that participation in mentoring programs contributed in 27% to their decision-making process (30). The low percentage influence of mentoring programs in this study results from the fact that 81% of the respondents were retired members and mentoring programs had only become available in the last few decades. But if women participated, they showed better exam results through participation in mentoring programs in the study Fallatah et al. and believe in better career planning through the mentoring program (31).

If the choice of specialization was a parameter for effective mentoring programs, women could become the focus of this consideration as they are still underrepresented in the surgical disciplines and there is a strong interest (93%; 607/653) in surgical mentoring programs.

Looking at the study period, it becomes apparent, that higher semesters, such as clinical studies and practical years, assign a high value to mentoring programs. Mentoring programs are important in all study periods but seem to become even more decisive with the higher semesters. Some studies presumed that students decide their speciality at the end of studies or after they have graduated from medical school (32) others claimed that they make decisions about their future medical careers during or even before medical school (33). Other studies showed that the majority of students in their last year have not yet decided on a subject (34, 35).

Interesting, however, was the high number of students interested in surgical specialties over all semester periods in this study. A previously reported declining interest in surgery during the course of studies (36–38) could not be observed in the gained data. The interest in surgical career remained rather constant, as other authors described, too (1, 39). Many factors influence the choice of specialization some, like family recommendations already exist before the study, but many others were recognized only by contact to a special field (40). The higher value of the mentoring programs in higher semesters could have many reasons, such as support for doctoral theses or exam preparation. Since the choice of career is also made individually in the course of medical studies, a mentoring program should also be individually accessible in each study period.

### Strength of the study

It is the first national study to look at peer mentoring programs at medical schools from the perspective of students and faculty, their availability, their usefulness, and their existence. Data from Germany were compiled by Meinel et al. published more than 10 years ago. These showed 22 mentoring programs at German medical universities and only investigated the presence of mentoring programs (5). No recent data have been collected since this study. The present study thus describes for the first time the views of students and medical faculties on mentoring in Germany and may form the basis for further studies. For the first time in German literature, the students’ perspective on mentoring is also examined in detail in this case issue. Comparable data have not yet been provided. Thus, despite the low response rate, the available data may have made a significant contribution to the area of mentoring.

### Limitations of the study

As a limitation, in particular, highly interested students may have responded earlier than undecided or already familiar with the topic, rather than students without any connection to the topic in the past. Participation rates increased with the semester, so many of the students have a good insight into the possibilities of choosing a field of study and may have already firmly chosen their field of study. Although there were significantly more women studying medicine, there is a male majority among the respondents, which may influence the data. The mentoring relationships were not recorded in more detail and qualitative (the type of mentoring relationship, mentorship goals) or quantitative (frequency of meetings, hierarchical level of the mentor-mentee relationship) criteria of the mentoring relationship were not asked. But by simplifying the questionnaire, it was not possible to ask about the modalities of mentoring programs. Therefore, this study cannot evaluate the availability, structure and design of mentoring programs.

## Conclusion

In conclusion, it can be mentioned that mentoring programs are able to influence career planning. However, only two-thirds of medical schools offer mentoring programs and only one-third have surgical involvement in mentoring programs. Becoming more active in this direction, providing mentoring programs and offering more surgical content can be a way to counteract the lack of surgical trainees.

Women in medical school participated less in mentoring programs, thus the women represent a suitable group for mentoring offers in order to interest them in surgical postgraduate training. Students in higher semesters more frequently requested participation in surgical mentoring programs, so especially undecided medical students could be inspired by surgical subject areas through the influence of mentoring programs.

## Data availability statement

The original contributions presented in this study are included in the article/Supplementary material, further inquiries can be directed to the corresponding author.

## Ethics statement

Written informed consent was obtained from the individual(s) for the publication of any potentially identifiable images or data included in this article.

## Author contributions

IG, ES, and SH conceived the research and designed the experiments. DB participated in the design and interpretation of the data. SH performed experiments and analysis. IG and SH wrote the manuscript and participated in the revisions of it. All authors read and approved the final manuscript.
