# Supplementary material for: Is student mentoring career-defining in surgical disciplines? A comparative survey among medical schools and medical students for mentoring programs
Source: Front Med (Lausanne). 2022 Nov 23;9:1008509. doi: 10.3389/fmed.2022.1008509 (PMC9726918; doi:10.3389/fmed.2022.1008509)
Supplement: Supplementary file 1 [file Data_Sheet_1.pdf]

Annex

Questionnaire for students:

Question 1: What gender are you?

Female
Male
Other

Question 2: Which semester are you in?

1 to 4 semesters (pre-clinic)
5 to 10 semesters (clinic)
11 to 12 semesters (practical year)
Free semester
Other

Question 3: Have you ever participated in a mentoring program?

Yes
No.

Question 4: If so, have you benefited?

Yes
No.

Question 5: Has the mentoring program positively supported your choice of subject?

Yes
No.

Question 6: Which discipline do you want to pursue later?

Operational area
Non-operational subject

Question 7: If an operative subject has been chosen, it is a subject of

Surgery
Other (e. g. gynaecology, urology, ENT. . .)

Question 8: If a surgical discipline is sought, what sub-specialization is sought?

General surgery

Visceral surgery
Orthopaedics/accident surgery
Plastic Surgery
Pediatric surgery
Neurosurgery
Oral-Jaw-Facial Surgery

Question 9: Would you participate in a structuring mentoring program in a surgical discipline?

Yes
No.

Question 10: Do you think it might inspire your choice of surgical discipline?

Yes
No.

Questionnaire for the Faculties of Medicine

1. How many students are enrolled at your medical school?

100 – 500
500 – 1000
1000 – 1500
1500 – 2000
2000 – 4000
4000 – 6000
>6000

2. Does the Faculty of Medicine offer student mentoring programs?

Yes
No
in planning
at an earlier point in time once

3. If so, how many students have already taken advantage of this student mentoring program?

0-50
50-100
100-200
200-500
500-1000
>1000

4. Are the student mentoring programs offered a structured program?

Yes
No

5. Are the structured programmes cross-clinical?

Yes
No

6. Which medical departments participate in the student mentoring program?

7. Are there criteria defined by the Faculty of Medicine/University regarding the structure, content and structure of the mentoring program?

Yes
No

8. If so, are these criteria designed to meet the standards set by the German Medical Association or similar standards?

Yes
No

9. Is there a specific budget or other funding sources to support the provision of student mentoring programs at your medical school?

no budget
1 to 5000 Euro
5000 to 10 000 Euro
10 000 to 20 000 euros
20 000 to 50 000 Euro
more than EUR 50 000

10. Would you like more support from relevant professional societies (DGIM/DGOU/GMA / GMDS etc.) and academic institutions or public bodies (DFG / BMBF) for the development and funding of structured mentoring programs for medical students?

yes
No

11. If so, what kind of support would you like to receive?

12. If there are currently no student mentoring programs offered at your medical school, do you think students would take advantage of such programs?

Yes
No
